# Grazing effects on the nutritive value of dominant species in steppe grasslands of northern China

**DOI:** 10.1186/s12898-018-0186-8

**Published:** 2018-09-03

**Authors:** Xiajie Zhai, Yingjun Zhang, Kun Wang, Qian Chen, Shuiyan Li, Ding Huang

**Affiliations:** Department of Grassland Science, College of Animal Science and Technology, China Agricultural University, Beijing, 100193 China

**Keywords:** Nutritive value, Forage, Grazing, Grassland

## Abstract

**Background:**

Forage nutritive value plays an important role in livestock nutrition and maintaining sustainable grassland ecosystems, and grazing management can affect the quality of forage. In this study, we investigated the effects of different grazing intensities on the nutritive values of *Leymus chinensis* (Trin.) Tzvelev, *Artemisia* spp. and *Carex duriuscula* C. A. Mey in the steppes of China during the growing seasons from 2011 to 2013. Five grazing management treatments were implemented: (1) rest grazing in spring, heavy grazing in summer and moderate grazing in autumn (RHM), (2) rest grazing in spring, moderate grazing in summer and heavy grazing in autumn (RMH), (3) heavy grazing though all seasons (HHH), (4) heavy grazing in spring and summer and moderate grazing in autumn (HHM) and (5) continuous moderate grazing in all seasons (MMM).

**Results:**

There were significant effects of year, season, treatment, and year × season and year × treatment interactions only on the crude protein of *L. chinensis* (P < 0.05). The crude protein concentrations of *L. chinensis* in the plots of constant high grazing pressure (HHH) and reduced grazing pressure in the last grazing stage (HHM) were higher than with deferred grazing (RMH and RHM, P < 0.05) in spring from 2011 to 2012. For *Artemisia* spp. and *C. duriuscula*, the crude protein concentration in HHH was higher than that in RMH (P < 0.05) in the summer of 2011. There were no significant differences (P > 0.05) for ether extract, neutral detergent fiber, acid detergent fiber and Ca concentration for any of the grasses in spring and summer from 2011 to 2013 under the different grazing management treatments.

**Conclusions:**

The nutritive value of *L. chinensis* was more responsive to grazing disturbance than *Artemisia* spp. and *C. duriuscula*, and heavy grazing maintained a relatively high crude protein content in all species. Seasonal and interannual seasonal differences in grazing management combinations were two of the most important factors in determining the variability of forage nutritive value, including crude protein, ether extract, neutral detergent fiber, acid detergent fiber and calcium, for *L. chinensis*, *Artemisia* spp. and *C. duriuscula*. We suggest that moderate grazing should be adopted to ensure the quality and yield of forage and promote the sustainable development of animal husbandry.

## Background

The Eurasian steppe is the largest contiguous terrestrial biome on Earth. The grasslands of China constitute an important global ecosystem [1]. Approximately 90% of the grassland area in China is considered to be degraded [2]. Grazing in arid and semiarid grassland ecosystems depends on local forage resources, and the nutritive value of forage plays an important role in livestock nutrition and maintaining sustainable production systems [3, 4]. Forage nutritive value affects forage utilization by herbivores. Higher nutritive value grasses have the potential to significantly increase milk or meat production and profitability of an expanding animal husbandry industry [5]. While many studies have focused on nutritive value of forage in planted or natural grasslands [6–10], especially in America and Europe, fewer studies have been conducted on the natural steppe under different grazing management treatments over a long period. Detailed information and reliable data on the effects of grazing management on forage nutritive value in the natural steppe are needed to improve sustainable grazing methods and help alleviate the overgrazing problem.

Grazing intensity and season of the year are likely to be the two most critical factors that affect forage nutritive value in natural grasslands. Grazing intensity affects plant species composition [11–14] and forage quality [15–17]. Additionally, high grazing intensity indirectly results in higher nutritive value forage as herbage consumed by animals is less mature [18], is of good quality and has continual regrowth during the growing season.

The main objectives of this study were to (1) evaluate the response of forage nutritive value, including crude protein (CP), ether extract (EE), neutral detergent fiber (NDF), acid detergent fiber (ADF) and calcium (Ca), to seasonal changes in grazing intensity, and (2) determine the interaction of season, year, and grazing management on the variation in forage nutritive value in the steppe grassland of China.

## Results

### The effects of grazing treatment, year, season and their interactions on forage nutritive value

The community biomass of different grazing management treatments in 2011 to 2013 is shown in Table 1. The biomass in summer was significantly higher than that in spring, and the biomass of rest grazing plots in spring was significantly higher than that in heavy grazing areas in spring (P < 0.05). Significant differences in forage nutritive values existed among seasons, years and grazing management treatments (Table 2). For CP concentration, *L. chinensis* was very responsive to treatments. There were significant effects of year, season, treatment, and year × season and year × treatment interactions only on the CP of *L. chinensis* (P < 0.05). The effect of season × treatment was marginally significant on the CP of *L. chinensis* (df = 4, F = 2.5, P = 0.054). The CP concentrations of *Artemisia* spp. and *C. duriuscula* were significantly influenced by season (df = 1, F > 8, P < 0.01) and treatment (df = 4, F > 6, P < 0.01). For EE content, *L. chinensis*, *Artemisia* spp. and *C. duriuscula* were all significantly influenced by the year (df = 2, F > 4, P < 0.05) but not the grazing management treatments. For NDF concentration, all plants were significantly influenced by season (df = 1, F > 6, P < 0.05), and *L. chinensis* was also affected significantly by grazing intensity (df = 4, F = 3.0, P < 0.05). Different seasons resulted in different ADF concentrations for all three species (*L. chinensis,* F = 11.1; *Artemisia* spp., F = 10.6; *C. duriuscula*, F = 27.1; P < 0.01). Furthermore, the ADF concentration of *C. duriuscula* was significantly influenced by year (df = 2, F = 4.9, P < 0.05). The forage Ca concentration was significantly affected by year and season for *L. chinensis* (year, df = 2, F = 26.0; season, df = 1, F = 18.1; P < 0.05) and *C. duriuscula* (year, df = 2, F = 11.6; season, df = 1, F = 8.2; P < 0.05). However, Ca concentration of *Artemisia* spp. was not significantly influenced by any of the factors (P > 0.05).

**Table 1:** The biomass of different grazing management treatments in 2011 to 2013

Grazing management treatments	Biomass (kg ha^−1^)					
	2011		2012		2013	
	Spring	Summer	Spring	Summer	Spring	Summer
RHM	818 (45)	2622 (151)	915 (52)	1810 (56)	897 (37)	2391 (103)
RMH	854 (36)	2708 (137)	850 (19)	2307 (63)	864 (41)	2372 (88)
HHH	285 (21)	872 (43)	476 (23)	509 (33)	542 (35)	1630 (61)
HHM	313 (22)	984 (51)	398 (19)	586 (41)	525 (37)	1714 (45)
MMM	532 (28)	1506 (72)	647 (34)	1010 (55)	984 (54)	2238 (99)

The numbers in parentheses indicate standard error

**Table 2:** Effects of various sources of variation on forage nutritive value

Source of variation	df	CP (crude protein)						EE (ether extract)						NDF (neutral detergent fiber)					
		*L. chinensis*		*Artemisia* spp.		*C. duriuscula*		*L. chinensis*		*Artemisia* spp.		*C. duriuscula*		*L. chinensis*		*Artemisia* spp.		*C. duriuscula*	
		F	P	F	P	F	P	F	P	F	P	F	P	F	P	F	P	F	P
Year	2	12.4	0.000	1.1	0.35	0.9	0.414	4.4	0.017	17.6	0.000	13.1	0.000	0.9	0.407	2.0	0.152	1.3	0.273
Season	1	23.9	0.000	8.8	0.004	12.2	0.001	0.4	0.55	1.5	0.231	0.5	0.488	13.0	0.001	15.2	0.000	6.7	0.012
Treatment	4	14.8	0.000	6.1	0.000	7.0	0.000	0.4	0.802	0.2	0.945	0.8	0.541	3.0	0.026	0.4	0.844	0.9	0.487
Year × season	2	4.2	0.020	0.1	0.931	0.2	0.826	0.1	0.878	0.6	0.558	0.3	0.721	1.0	0.38	0.2	0.816	0.1	0.943
Year × treatment	8	4.2	0.001	0.5	0.817	1.8	0.092	1.7	0.127	0.4	0.908	0.5	0.821	1.2	0.332	0.5	0.845	0.8	0.624
Season × treatment	4	2.5	0.054	0.3	0.872	0.1	0.971	1.0	0.393	0.4	0.807	0.4	0.833	0.2	0.938	0.6	0.666	0.6	0.645
Year × season × treatment	8	1.0	0.442	0.3	0.974	1.3	0.238	0.8	0.636	0.9	0.525	0.3	0.961	1.6	0.138	0.6	0.772	0.9	0.516

Source of variation	df	ADF (acid detergent fiber)						Ca (calcium)					
		*L. chinensis*		*Artemisia* spp.		*C. duriuscula*		*L. chinensis*		*Artemisia* spp.		*C. duriuscula*	
		F	P	F	P	F	P	F	P	F	P	F	P
Year	2	1.8	0.178	1.7	0.189	4.9	0.011	26.0	0.000	1.2	0.297	11.6	0.000
Season	1	11.1	0.001	10.6	0.002	27.1	0.000	18.1	0.000	1.3	0.257	8.2	0.006
Treatment	4	1.4	0.242	1.4	0.247	0.5	0.717	1.3	0.263	0.6	0.644	1.1	0.358
Year × season	2	0.7	0.518	0.6	0.536	1.6	0.214	1.9	0.155	0.1	0.871	0.9	0.403
Year × treatment	8	1.7	0.129	1.2	0.32	0.6	0.81	1.2	0.33	1.1	0.347	0.3	0.961
Season × treatment	4	0.5	0.76	1.0	0.42	0.3	0.893	0.6	0.69	0.9	0.491	0.7	0.567
Year × season × treatment	8	0.4	0.927	0.6	0.813	0.8	0.601	1.0	0.447	1.2	0.343	0.8	0.57

P represents probability values for significant differences; DF is the degrees of freedom

### Forage nutrient concentration under different grazing management treatments

**Fig. 1:**
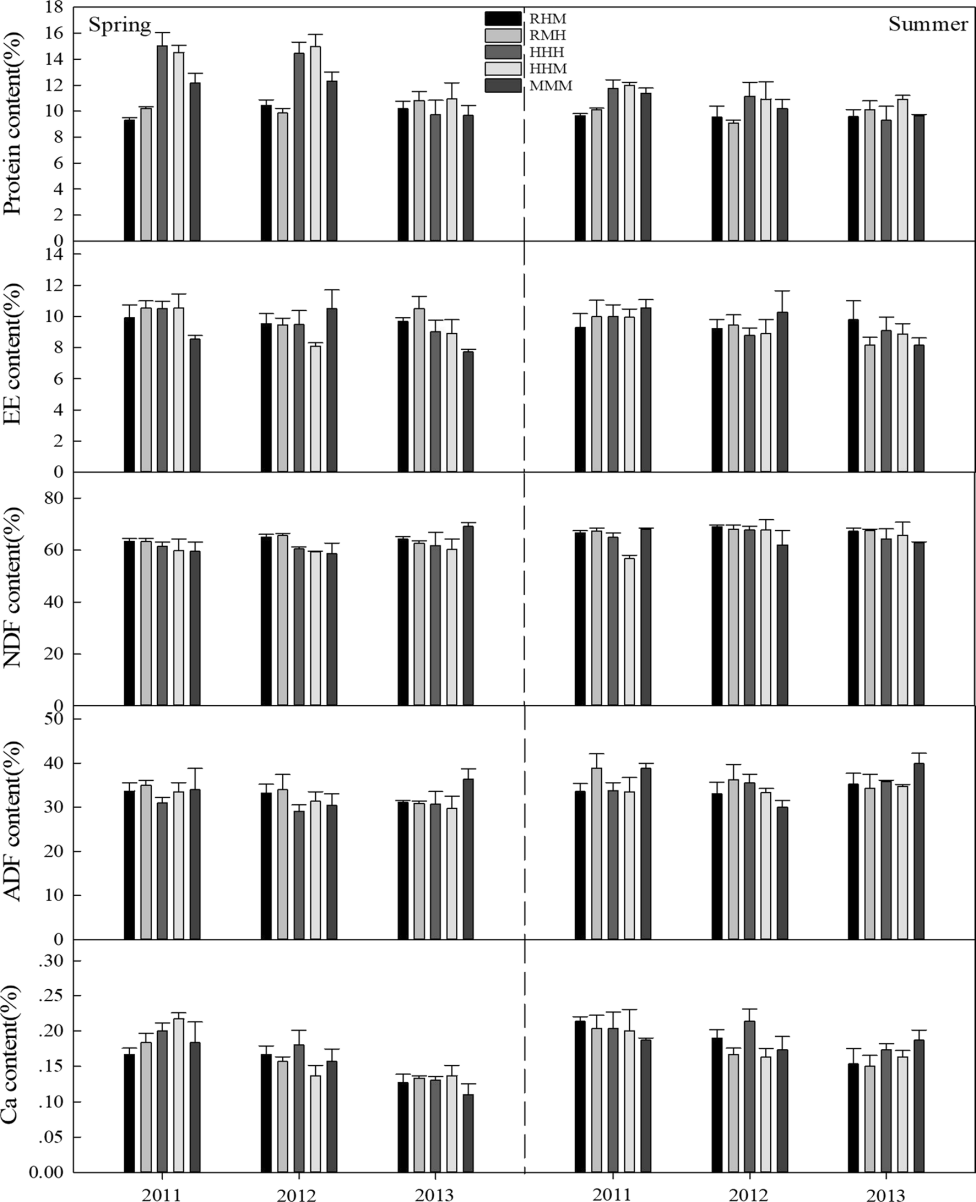
The nutrient concentration (%) of *L. chinensis* under different grazing management treatments in typical steppe grassland. Error bars indicate standard error

**Fig. 2:**
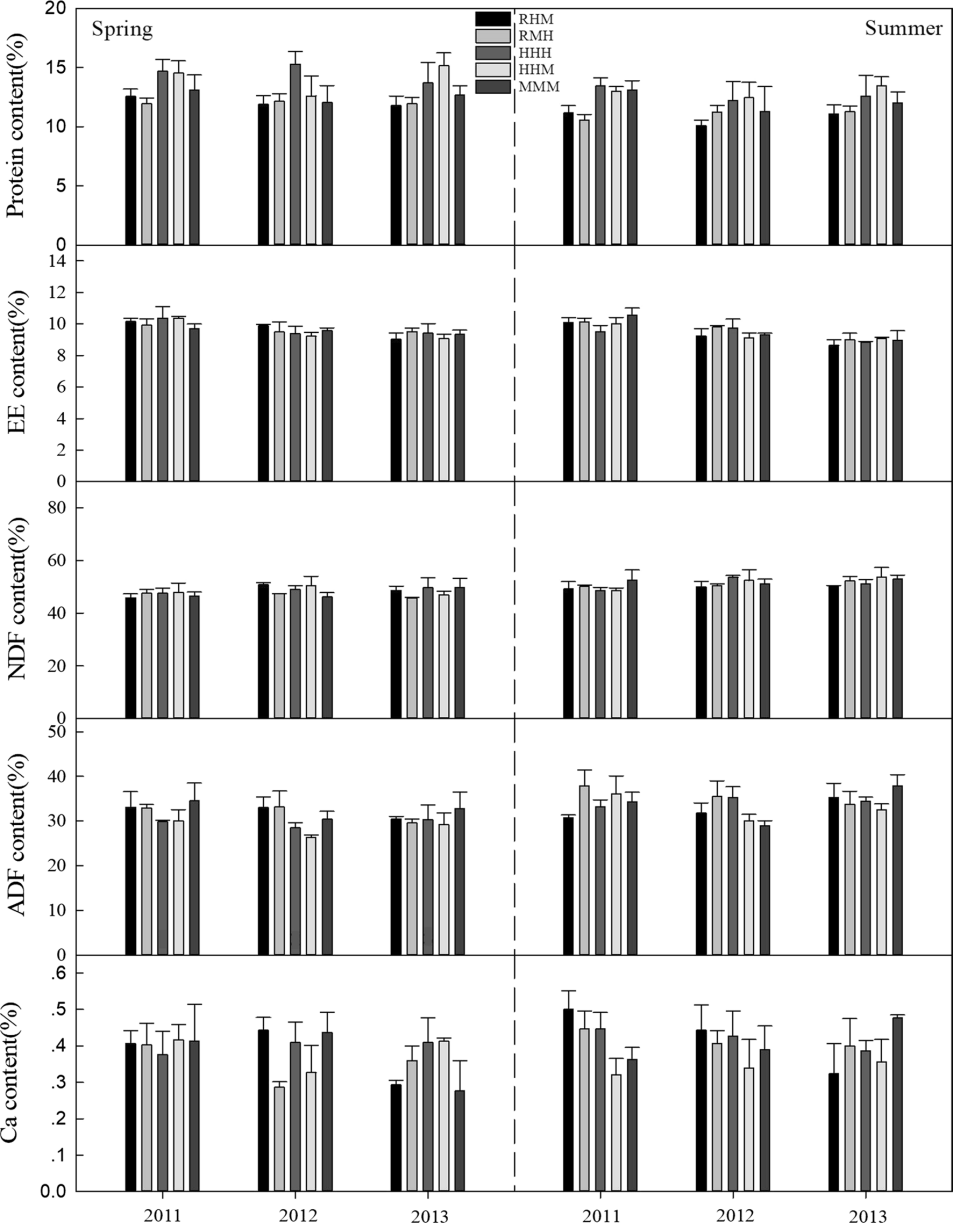
The nutrient concentration (%) of *Artemisia* spp. under different grazing management treatments in typical steppe grassland. Error bars indicate standard error

**Fig. 3:**
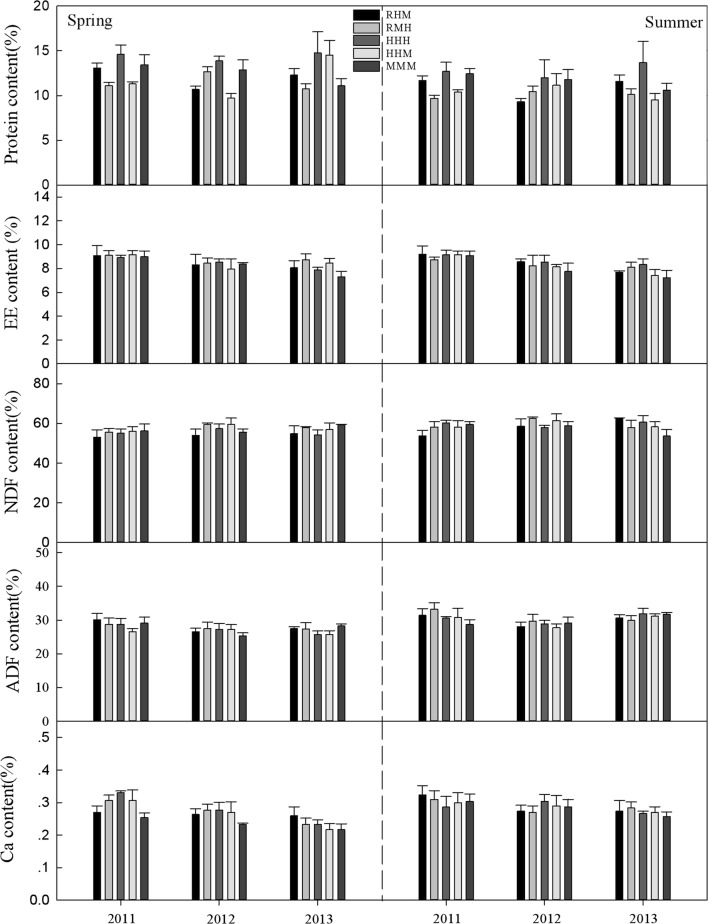
The nutrient concentration (%) of *C. duriuscula* under different grazing management treatments in typical steppe grassland. Error bars indicate standard error

The CP concentrations of *L. chinensis* under the treatments of heavy grazing though all seasons (HHH) and heavy grazing in spring and summer and moderate grazing in autumn (HHM) were higher than in the plots of rest grazing in spring, heavy grazing in summer and moderate grazing in autumn (RMH) and rest grazing in spring, moderate grazing in summer and heavy grazing in autumn (RHM) in spring from 2011 to 2012 (P < 0.05), with continuous moderate grazing in all seasons (MMM) being intermediate (Fig. 1). The highest and lowest concentrations were 15.0% (HHH) and 9.0% (RMH), respectively. Similarly, during the summer of 2011, the concentrations of 12.0% and 11.7% in HHM and HHH, respectively, were higher than the 9.6% in RMH. However, the differences were not significant in 2013 and the summer of 2012 (P > 0.05). For *Artemisia* spp. and *C. duriuscula* (Figs. 2, 3), the CP concentration in HHH was higher than that in RMH (P < 0.05) in the summer of 2011. There were no significant differences (P > 0.05) for EE, NDF, ADF and Ca concentration for any of the grasses in the spring and summer from 2011 to 2013 under the different grazing management treatments.

## Discussion

Forage CP concentration is an important indicator of forage nutritive value, and good forage quality is generally associated with high CP and low fiber [19, 20]. Ma et al. [21] reported that the forage community had higher CP but lower NDF and ADF when grazed using HHM and HHH compared with RHM, RMH and MMM (P < 0.05). In the current study, the results focused on the most important forage species, and our data revealed that spring grazing and a higher stocking rate in this period increased the forage quality because grazing removed old material leaving fresh regrowth. Forage CP concentration increased sharply and NDF decreased as stocking rate increased [22], and a similar conclusion was reached from the data in the current study.

Shi et al. [23] found that the Tibetan grassland had higher quality forage than the Inner Mongolian grasslands, and alpine meadows had the best quality forage because of the high CP concentration of the meadow forage. Various factors, including climate, plant species, livestock species and grazing methods, determine the occurrence of high CP concentration. In addition, our results were consistent with several previous studies showing that changes in management intensity could affect nutritive value of the forage [7, 24–27] and that forage nutritive value was enhanced by intensive grazing [18, 28], including linear increases in CP with increasing grazing intensity.

Because of continuous heavy grazing and the fact that animals select forage species of the highest quality available at any point in time [29], grasslands grazed intensively have been characterized by relatively less mature regrowth, and due to frequent grazing, the maturation and lignification processes are decelerated [30]. Sheep were the only species in this study and may provide an additional explanation for the result. If cattle were grazed together with sheep, then the strategies for mutual survival in a heterogeneous environment with high variation in quality of forage species would differ greatly [31].

## Conclusions

The nutritive value of *L. chinensis* was more responsive to grazing disturbance than *Artemisia* spp. and *C. duriuscula*. Heavy grazing maintained a relatively high crude protein content in all species compared to other grazing intensities. Seasonal and interannual seasonal differences were two of the most important factors in determining the variability of forage nutritive value, including CP, EE, NDF, ADF and Ca for *L. chinensis*, *Artemisia* spp. and *C. duriuscula*. We suggest that moderate grazing should be adopted to ensure the quality and yield of forage and promote the sustainable development of animal husbandry.

## Methods

### Study site description

The study was conducted at the Guyuan site of the State Key Monitoring and Research Station of Grassland Ecosystems, in Hebei Province of the People’s Republic of China (41°44ʹ N, 115°40ʹ E). The site has an elevation of 1380 m and is a typical steppe which had been fenced to exclude grazing since 2004 because of grassland degradation [32]. Average annual precipitation is approximately 430 mm, of which 80% falls in the growing season (June to August). The average precipitation from June to October 2011, 2012 and 2013 were 238, 261 and 378 mm, respectively. The local climate is temperate terrestrial monsoon with a frost-free period of 85–95 days [33]. January is the coldest month with an average temperature of − 18.6 °C, while July is the warmest month with an average temperature of 17.6 °C. The average temperature in the growing seasons (from June to October) of 2011, 2012 and 2013 were 12.7, 12.4 and 13.6 °C, respectively. The grassland is dominated by *Leymus chinensis*, and the growing season extends from May to the end of September based on the phenology of dominant and common species and long-term seasonal patterns of temperature and precipitation in this area. The soil is a Calcic-orthic Aridisol. Average organic matter concentration in the 0–10 cm soil layer is 3.65% and total N is 0.16%.

### Design of the grazing experiment

**Fig. 4:**
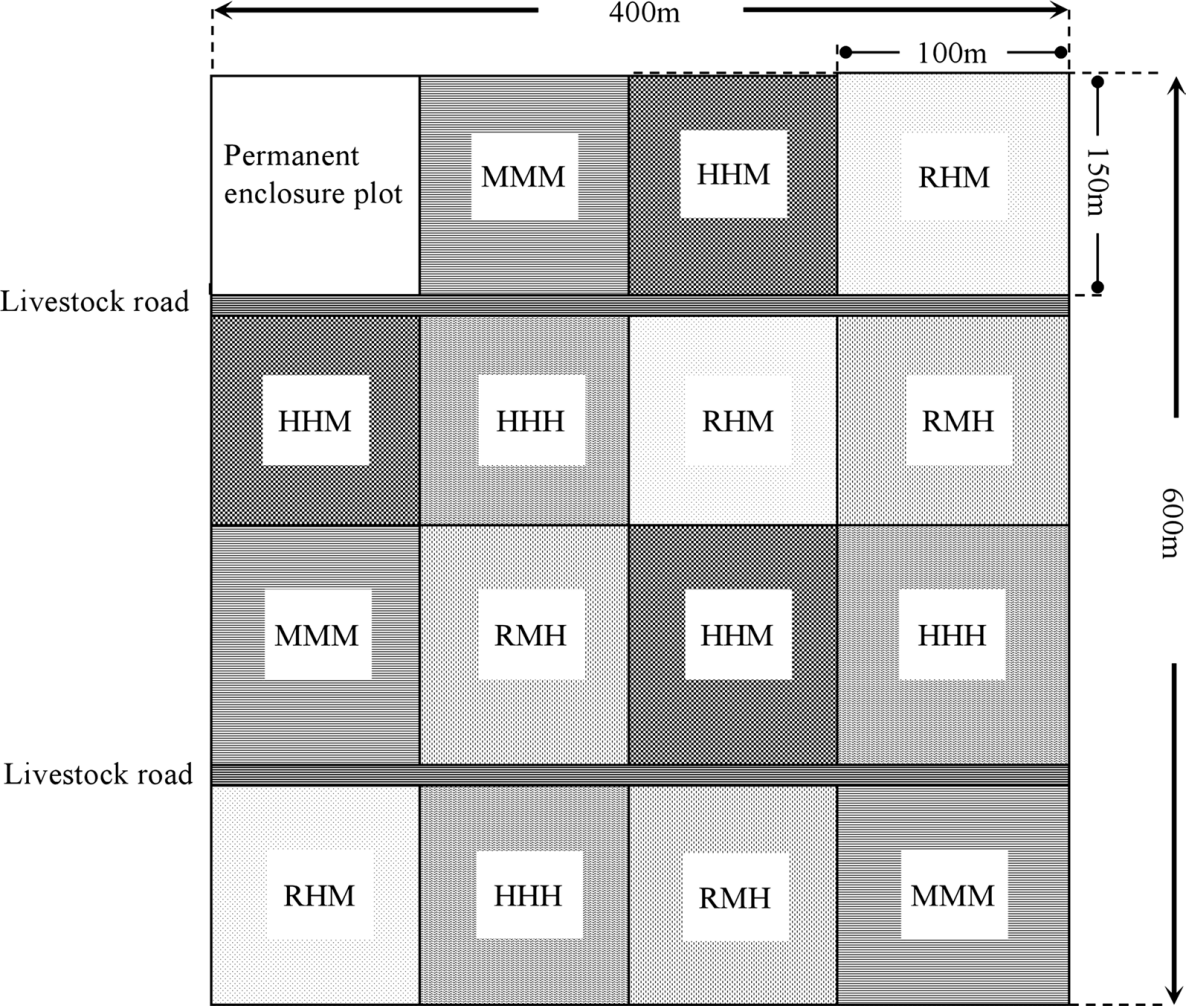
Experimental plot layout

The grazing management treatments were conducted from 2011 to 2013. The treatments in this grazing experiment involved combinations of rest grazing (0 sheep ha^−1^) (RG), moderate grazing (6.7 sheep ha^−1^) (MG) and heavy grazing (9.3 sheep ha^−1^) (HG). Five grazing management treatments were implemented: (1) RG in spring, HG in summer and MG in autumn (RHM), (2) RG in spring, MG in summer and HG in autumn (RMH), (3) HG though all seasons (HHH), (4) HG in spring and summer and MG in autumn (HHM) and (5) continuous MG in all seasons (MMM). There were 3 replicates for each treatment in a completely randomized design with a total of 15 plots, each 1.5 ha in size (Fig. 4). A core group of animals remained within each treatment throughout the year, and new animals were used each year [34].

At the beginning of each year, Mongolian sheep (< 2 years old) of uniform live weight (average starting weight 36–39 kg) were randomly allocated to the 15 experimental plots. Stocking rates for heavy grazing were the district average and moderate grazing was 30% less. Ten sheep were assigned to moderate stocking rate plots and fourteen were assigned to heavy stocking rate plots from 2011 to 2012, which was reduced to seven and eleven sheep in 2013, respectively, as it was apparent that grazing pressure was high in the two previous years resulting in very high utilization of aboveground vegetation [34]. Spring is when dominant species begin to grow until they flower. Summer is the active growth period until plants have finished flowering and have set seed. Autumn is when plant growth terminates because of an abrupt decrease in temperature and precipitation [32]. The length of grazing for each season varied from 29 to 30 days in spring, 47–60 days in summer and 25–32 days in autumn, depending upon the year. In 2011, the spring period was from June 9 to July 12, the summer period was July 13 to September 10, and the autumn period was September 11 to October 10. In 2012, the spring period was from June 15 to July 14, the summer period was July 15 to September 4, and the autumn period was September 5 to September 26. In 2013, the spring period was from June 21 to July 20, the summer period was July 21 to August 16, and the autumn period was August 17 to September 8. Animal numbers were added or removed throughout the year as required to apply grazing management treatments [34]. Sheep were penned at night in each experimental unit for them to rest without grazing. Water and salt were available at all times and no supplement was provided.

### Measurement of forage nutritive values

*Leymus chinensis*, *C. duriuscula*, *and Artemisia* spp. were the most common plant species and the main forage for grazing animals in this area. Five sampling quadrats (1 m × 1 m) were randomly established in each plot. The aboveground forage was cut using hand shears and collected in June 25–26 and August 10–11 from 2011 to 2013. Each species was collected separately. Each sample was a composite of 5 sampling quadrats and litter was not included in the forage sample. Collected samples were dried in an oven to constant weight at 60 °C and ground using a ball mill (NM200, Retsch, Germany) prior to laboratory analyses. Forage quality was estimated using a proximate analysis system (Weende system), which divided the dry matter into crude protein (CP) and ether extract (EE) [35]. Mineral calcium concentration was measured using a spectrophotometer at 430 nm. Neutral detergent fiber (NDF) and acid detergent fiber (ADF) were determined sequentially by an ANKOM 200 Fiber Analyzer (Ankom Technology, Macedon, NY, USA) and analyzed using the method of Van Soest et al. [36]. The analysis of forage quality was conducted in the laboratory of the China Agricultural University, Beijing.

### Statistical analysis

Forage nutritive value data were analyzed using repeated measures in SPSS with grazing treatment, season, and year as well as their interactions as fixed factors, and plot considered a random effect. Plot was treated as a repeated measure variable. The statistical model used was as follows:

$${\text{y}}_{\text{rijk}} = \,\upmu + {\text{T}}_{\text{i}} + {\text{S}}_{\text{j}} + {\text{Y}}_{\text{k}} + {\text{TS}}_{\text{ij}} + {\text{TY}}_{\text{ik}} + {\text{SY}}_{\text{jk}} + {\text{TSY}}_{\text{ijk}} + {\text{P}}_{\text{r}} + {\text{e}}_{\text{rijk}}$$

where y_rijk_ is the response in year k (k = 1–3) for season j (j = 1–2) in treatment group i (i = 1–5) in plot r (r = 1–3); µ is the overall mean; T_i_ is the fixed effect of treatment i; S_j_ is the fixed effect of season j; Y_k_ is the fixed effect of year k; TS_ij_ is the fixed interaction effect of treatment i with season j; TY_ik_ is the fixed interaction effect of treatment i with year k; and SY_jk_ is the fixed interaction effect of season j with year k. TSY_ijk_ is the fixed interaction effect of treatment i with season j for year k. P_r_ is the plot replicate; and e_rijk_ is the random error for year k for season j in treatment group i. Significant differences in treatment means were determined using the Tukeyʼs test with the level of significance of P < 0.05.

## References

[1] Bai Y, Wu J, Pan Q, Huang J, Wang Q, Li F (2007). Positive linear relationship between productivity and diversity: evidence from the Eurasian Steppe. J Appl Ecol.

[2] Tong C, Wu J, Yong S, Yang J, Yong W (2004). A landscape-scale assessment of steppe degradation in the Xilin River Basin, Inner Mongolia, China. J Arid Environ.

[3] DeYoung RW, Hellgren EC, Fulbright TE, Robbins WF, Humphreys ID (2000). Modeling nutritional carrying capacity for translocated desert bighorn sheep in western Texas. Restor Ecol.

[4] Briske DD, Derner JD, Brown JR, Fuhlendorf SD, Teague WR, Havstad KM (2008). Rotational grazing on rangelands: reconciliation of perception and experimental evidence. Rangeland Ecol Manag.

[5] Taube F, Gierus M, Hermann A, Loges R, Schönbach P (2014). Grassland and globalization—challenges for north-west European grass and forage research. Grass Forage Sci.

[6] Marten GC, Sheaffer CC, Wyse DL (1987). Forage nutritive value and palatability of perennial weeds. Agron J.

[7] Schönbach P, Wan H, Schiborra A, Gierus M, Bai Y, Müller K (2009). Short-term management and stocking rate effects of grazing sheep on herbage quality and productivity of Inner Mongolia Steppe. Crop Pasture Sci.

[8] Pelletier S, Tremblay GF, Bélanger G, Bertrand A, Castongay Y, Pageau D (2010). Forage nonstructural carbohydrates and nutritive value as affected by time of cutting and species. Agron J.

[9] Henkin Z, Ungar ED, Dvash L, Perevolotsky A, Yehuda Y, Sternberg M (2011). Effects of cattle grazing on herbage quality in a herbaceous Mediterranean rangeland. Grass Forage Sci.

[10] Allen E, Sheaffer C, Martinson K (2013). Forage nutritive value and preference of cool-season grasses under horse grazing. Agron J.

[11] Belesky DP, Feldhake CM, Boyer DG (2002). Herbage productivity and botanical composition of hill pasture as a function of clipping and site features. Agron J.

[12] Li Y, Wang W, Liu Z, Jiang S (2008). Grazing gradient versus restoration succession of *Leymus chinensis* (Trin.) Tzvel. grassland in Inner Mongolia. Restor Ecol.

[13] Zhao W, Chen SP, Han XG, Lin G (2009). Effects of long-term grazing on the morphological and functional traits of *Leymus chinensis* in the semiarid grassland of Inner Mongolia, China. Ecol Res.

[14] Ren H, Schönbach P, Wan H, Gierus M, Taube F (2012). Effects of grazing intensity and environmental factors on species composition and diversity in typical steppe of Inner Mongolia, China. PLoS ONE.

[15] Belesky DP, Fedders JM, Turner KE, Ruckle JM (1999). Productivity, botanical composition, and nutritive value of swards including forage chicory. Agron J.

[16] Skinner RH, Gustine DL, Sanderson MA (2004). Growth, water relations, and nutritive value of pasture species mixtures under moisture stress. Crop Sci.

[17] Deak A, Hall MH, Sanderson MA (2009). Grazing schedule effect on forage production and nutritive value of diverse forage mixtures. Agron J.

[18] Nelson CJ (2012). Conservation outcomes from pastureland and hayland practicess: assessment, recommendations, and knowledge gaps.

[19] Van Soest PJ (1994). Nutritional ecology of the ruminant.

[20] Barnes RF, Nelson CJ, Collins M, Moore KJ (2003). Forages: an introduction to grassland agriculture.

[21] Ma L, Yuan F, Liang H, Rong YP (2014). The effects of grazing management strategies on the vegetation, diet quality, intake and performance of free grazing sheep. Livest Sci.

[22] Sasaki T, Ohkuro T, Jamsran U, Takeuchi K (2012). Changes in the herbage nutritive value and yield associated with threshold responses of vegetation to grazing in Mongolian rangelands. Grass Forage Sci.

[23] Shi Y, Ma YL, Ma WH, Liang C, Zhao X, Fang J (2013). Large scale patterns of forage yield and quality across Chinese grasslands. Chin Sci Bull.

[24] White TA, Barker DJ, Moore KJ (2004). Vegetation diversity, growth, quality and decomposition in managed grasslands. Agric Ecosyst Environ.

[25] Cozzi G, Gottardo F (2005). Feeding behaviour and diet selection of finishing Limousin bulls under intensive rearing system. Appl Anim Behav Sci.

[26] Marriott CA, Bolton GR, Fisher JM, Hood K (2005). Short-term changes in soil nutrients and vegetation biomass and nutrient content following the introduction of extensive management in upland sown swards in Scotland, UK. Agric Ecosyst Environ.

[27] Pavlů V, Hejcman M, Pavlů L, Gaisler J, Nežerková P (2006). Effect of continuous grazing on forage quality, quantity and animal performance. Agric Ecosyst Environ.

[28] Garay AH, Sollenberger LE, Staples CR, Pedreira CGS (2004). ‘Florigraze’and ‘Arbrook’rhizoma peanut as pasture for growing Holstein heifers. Crop Sci.

[29] Sebata A, Ndlovu LR (2012). Effect of shoot morphology on browse selection by free ranging goats in a semi-arid savanna. Livest Sci.

[30] Milchunas DG, Varnamkhasti AS, Lauenroth WK, Goetz H (1995). Forage quality in relation to long-term grazing history, current-year defoliation, and water resource. Oecologia.

[31] Selemani IS, Eik LO, Holand Ø, Adnoy T, Mtengeti E, Mushi D (2013). Variation in quantity and quality of native forages and grazing behavior of cattle and goats in Tanzania. Livest Sci.

[32] Chen WQ, Huang D, Liu N, Zhang YJ, Badgery WB, Wang XY (2015). Improved grazing management may increase soil carbon sequestration in temperate steppe. Sci Rep.

[33] Huang D, Wang K, Wu WL (2007). Dynamics of soil physical and chemical properties and vegetation succession characteristics during grassland desertification under sheep grazing in an agro-pastoral transition zone in Northern China. J Arid Environ.

[34] Zhang YJ, Huang D, Badgery WB, Kemp DR, Chen WQ, Wang XY (2015). Reduced grazing pressure delivers production and environmental benefits for the typical steppe of north China. Sci Rep.

[35] Zhang LY (2007). Analysis of feed and assay methodology of feed quality.

[36] Van Soest PJ, Robertson JB, Lewis BA (1991). Methods for dietary fiber, neutral detergent fiber, and nonstarch polysaccharides in relation to animal nutrition. J Dairy Sci.

